# On Contagion

**Published:** 1816-04

**Authors:** 


*For the London Medical and Physical Journal.*

### *On Contagion;* by an Army Surgeon,

IF my knowledge of the late communications upon the subject of Precautions against Contagion is not deficient, I believe that the means employed in places where pestilential diseases are prevailing, consists, at present, in attention attention to cleanliness, and destroying of contagious effluvia by fumigations. I wish, therefore, to submit it to the judgment of the Editors of the Medical and Physical Journal, whether or not there is room to offer the suggestion of a preventitive medicine in addition to the above means. Such could, perhaps, be no where better sought after than in the antiseptic property of the mineral acids. A palatable drink or linctus made with the muriatic acid, and taken daily in any quantity under what may affect the health, might be proposed with this view; and it is probable that the use of the mineral acids internally would be found to have a greater influence in preserving health in infected places and quarantines than any external means whatsoever.

*Jersey; Jan.* 1816.

